# Controllable switching between planar and helical flagellar swimming of a soft robotic sperm

**DOI:** 10.1371/journal.pone.0206456

**Published:** 2018-11-02

**Authors:** Islam S. M. Khalil, Ahmet Fatih Tabak, Mohamed Abou Seif, Anke Klingner, Metin Sitti

**Affiliations:** 1 Mechatronics Department, The German University in Cairo, New Cairo, Egypt; 2 Physics Department, The German University in Cairo, New Cairo, Egypt; 3 Physical Intelligence Department, Max Planck Institute for Intelligent Systems, Stuttgart, Germany; Texas A&M University System, UNITED STATES

## Abstract

Sperm cells undergo a wide variety of swimming patterns by a beating flagellum to maintain high speed regardless of the rheological and physical properties of the background fluid. In this work, we develop and control a soft robotic sperm that undergoes controllable switching between swimming modes like biological sperm cells. The soft robotic sperm consists of a magnetic head and an ultra-thin flexible flagellum, and is actuated using external magnetic fields. We observe that out-of-plane wobbling of the head results in helical wave propagation along the flagellum, whereas in-plane wobbling achieves planar wave propagation. Our theoretical predictions and experimental results show the ability of the soft robotic sperm to change its swimming speed by tuning the beating frequency of its flagellum and the propulsion pattern. The average speed of the soft robotic sperm increases by factors of 2 and 1.2 in fluids with viscosity of 1 Pa.s and 5 Pa.s at relatively low actuation frequencies, respectively, when they switch between planar to helical flagellar propulsion.

## Introduction

Sperm is a self-motile cell with approximately 40-50 μm in length. Different beat patterns of sperm cells can be observed by varying the viscosity of the background fluid [1, 2]. For example, the spermatozoa of *Echinus esculentus* execute transitions between planar and helical flagellar propulsion based on the external viscosity. Woolley and Vernon have demonstrated that increasing the viscous load on the cell results in a transition from planar to helical flagellar propulsion, while a much greater increase in viscosity achieves a reversion from helical to quasi-planar flagellar propulsion [3]. These swimming patterns have inspired researchers to design and fabricate externally actuated robots at micro and nano-scales to realize a single swimming pattern only [4, 5]. In contrast to sperm cells that modify their swimming patterns autonomously, man-made robots require an external intervention to modify the deformation of their soft bodies. Nevertheless, sperm cells provide significant inspiration for several research groups. For instance, planar flagellar propulsion has been experimentally demonstrated by Dreyfus *et al*. using a chain of magnetic particles coated with streptavidin and attached to a blood cell to resemble the flagellum and head of a sperm cell [6]. Pak *et al*. have also presented a flexible nanowire motor driven by a magnetic field, and consists of a 1.5 mm-long Ni head and a 4 mm-long flexibe Ag tail [7]. Williams *et al*. have also demonstrated planar flagellar propulsion in low Reynolds numbers using a biohybrid swimmer that consists of a polydimethylsiloxane filament actuated by a cluster of contractile cells [8]. Swimming using multiple flexible flagella [9, 10], propulsion using continuous undulatory deformation of a distributed magnetization profile of a flexible sheet [11], and point-to-point motion control of a sperm-shaped microrobot have been demonstrated using controlled planar flagellar propagation [12]. Several designs of microrobots have also been proposed to achieve helical flagellar propulsion in low Reynolds numbers [13]. Ghosh and Fischer have demonstrated motion control of chiral colloidal propellers using homogenous magnetic fields [14]. In contrast to propulsion using planar flagellar wave propagation, helical propulsion has been transferred into *in vitro* trials and several nanotechnology applications [15]–[22]. For instance, Servant *et al*. have tracked the controlled navigation of a swarm of functionalized artificial bacterial flagella in the peritoneal cavity of a mouse [20]. Very recently, Medina-Sanchez *et al*. have employed helical microrobot driven using rotating magnetic fields to assist immotile sperm cells during *in vitro* fertilization [23]. It follows from the mentioned brief review that propulsion using planar and helical flagellar wave propagation have not been simultaneously demonstrated using the same microrobot. The capability to achieve switching between planar to helical and reversion from helical to planar has not yet been demonstrated for mainly two reasons: Rigidity of the microrobot and fixed orientation of its magnetization.

**Fig 1:**
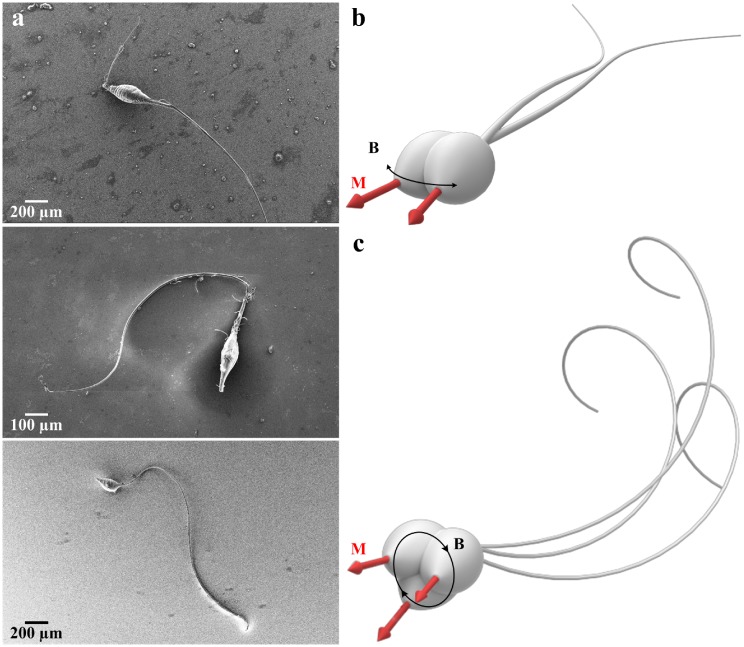
Soft robotic sperm switches between planar to helical flagellar propulsion under the influence of controlled magnetic fields. (a) Scanning electron microscopy images of the robotic sperm indicate similar morphology to that of biological sperm cells. The robotic sperm consists of a magnetic head and a flexible flagellum, and is fabricated using polystyrene. Magnetic particles are impeded into the head to provide magnetization (M). (b) Planar flagellar propulsion is achieved by applying in-plane uniform field (B) along direction of motion with a sinusoidally varying orthogonal component. (c) Wobbling of the head of the robotic sperm is achieved by applying out-of-plane field.

Switching between propulsion modes necessitates fabrication of soft microrobots with a controllable deformation pattern. This fabrication requirement has been demonstrated by Huang *et al*. by integrating self-folded hydrogel tubes to artificially approximate bacterial flagella and enhance the overall motility of soft microrobots [24]. Lum *et al*. have also proposed a universal programming methodology to achieve desired magnetization profiles for soft materials and enable realization of time varying shapes [25]. Huang *el al*. have presented a microfabrication technique to fabricate soft microrobots from thermoresponsive magnetic hydrogel nanocomposites [26]. Recently, biomimetic undulations of photoactive liquid-crystal elastomers have been achieved using structured monochromatic light by Palagi *et al*. [27]. An electrospinning and a DNA-based assembly techniques have been also presented to fabricate soft microrobots [28]. In this work, motivated by the unique ability of sperm cells to switch between planar and helical flagellar propulsion, we perform a combined theoretical and experimental investigation of the dynamics of soft robotic sperm that undergo controllable switching between these swimming patters. We investigate the ability of a soft robotic sperm (Fig 1a) to switch between planar (Fig 1b) and helical flagellar (Fig 1c) propulsion to maintain relatively high swimming speed in mediums with different viscosities. First, we develop a hydrodynamic model of the soft robotic sperm based on the resistive-force theory (RFT) to study the behaviour of the robot during each swimming mode and the influence of rheological properties of the medium [29, 30]. Second, we compare our theoretical predictions to experimental results using a soft robotic sperm actuated via controlled magnetic fields in millitesla range.

## Governing equations of the soft robotic sperm

**Fig 2:**
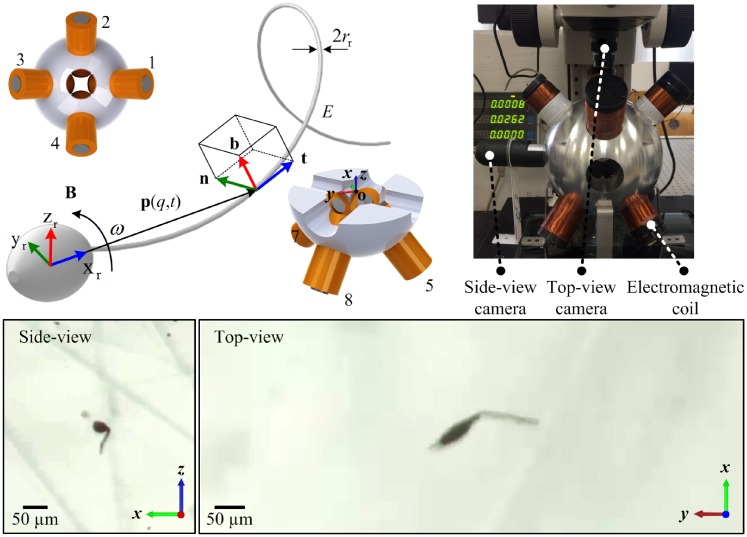
A configuration of 8 electromagnetic coils is used to generate periodic magnetic field B at frequency *ω*. Motion and deformation of the tail are observed using side- and top-view cameras and microscopic lenses. The soft robotic sperm is contained inside a deep chamber of silicone oil with viscosity of 1 Pa.s and 5 Pa.s. The chamber is located at the common center of the eight coils. The robot has tail diameter 2*r*_*t*_, length *l*_*t*_, and modulus of elasticity *E*. **t**, **n**, and **b** represent the tangent, normal, and bi-normal directions of the moving Frenet-Serret frame.

The robotic sperm used in this study is an electrospun beaded fiber (see Experimental section) with an elliptical magnetic head and flexible ultra-thin tail that resembles the morphology of a sperm cell, as shown in Fig 1a. The head has an average magnetic moment **M**, lying along the major axis of the head. This head is rigidly attached to an ultra-thin tail, of diameter 2*r*_t_, length *l*_t_, and modulus of elasticity *E*. Fig 2 shows the robotic sperm and the electromagnetic coils. We describe the shape of the ultra-thin tail at a given time *t* by the position vector **p**(*q*, *t*) of points along the centerline of the tail, where *q* represents the generalized coordinate of an arbitrary point along the long axis of the head of the robotic sperm. We express **p**(*q*, *t*) with respect to the material frame of the robotic sperm (**x**_r_, **y**_r_, **z**_r_), as shown in Fig 2, and is given by

$$\begin{array}{c} p\left(q,t\right)=[\begin{array}{ccc} q & \varphi_{y}\left(q,t\right) & \varphi_{z}\left(q,t\right) \end{array}], \end{array}$$ (1)

where *φ*_*y*_(*q*, *t*) and *φ*_*z*_(*q*, *t*) represent the deformation along **y**_r_- and **z**_r_-axis, respectively. With this vector, the moving Frenet-Serret frame can be expressed as follows:

$$\begin{array}{c} t=\frac{dp(q,t)/dq}{∥dp(q,t)/dq∥}\;\;,\;\;n=\frac{dt/dq}{∥dt/dq∥}\;\;,\;\;b=t\times n, \end{array}$$ (2)

where **t**, **n**, and **b** are the unit tangent, normal, and bi-normal vectors of the moving Frenet-Serret frame (Fig 2), respectively. The robotic sperm is externally actuated at frequency *ω* by a periodic magnetic field **B**, in its frame of reference via 8 computer-controlled electromagnetic coils. The following two patterns of magnetic fields are applied to induce planar and helical wave propagation along the flexible tail: first, planar wave propagation is achieved by periodic magnetic field constrained to a plane (Fig 1b); second, periodic out-of-plane fields forcing the magnetic head to follow a finite steradian to induce helical wave propagation along the elastic tail (Fig 1c). As the robot exhibits one of these patterns, the elastohydrodynamics of the elastic tail is responsible for the resistive-forces that act locally on the elastic tail due to the induced flow field, while immersed in a viscous fluid with density *ρ* and viscosity *μ*, characterized by low Reynolds number ($Re=\frac{\rho |V_{r}|L}{\mu }$) hydrodynamics on the order of $O(10^{-5})$, where *L* and **V**_r_ are the length and linear velocity of the robot in its frame of reference, respectively. The planar or helical wave propagation along the tail is achieved via magneto-elastic coupling between the tail and the magnetic torque generated by the magnetic field. The propagation of these waves along the tail is responsible for the propulsion with translational velocity **V**_r_ and rotational velocity **Ω**_r_. We assume small local deformations and use local rotations based on Frenet-Serret coordinates (Fig 2). The wave propagation is approximated by the Timoshenko-Rayleigh beam elements [31, 32]. Therefore, the governing equation along the lateral directions is given by

$$\begin{array}{c} EI\frac{\partial^{4}\varphi_{i}\left(q,t\right)}{\partial q^{4}}+\rho_{r}A\frac{\partial^{2}\varphi_{i}\left(q,t\right)}{\partial q^{2}}-\xi \frac{\partial^{4}\varphi_{i}\left(q,t\right)}{\partial q^{2}\partial t^{2}}+\frac{\rho_{r}^{2}AR_{g}^{2}}{\kappa G}\frac{\partial^{2}\varphi_{i}\left(q,t\right)}{\partial t^{2}}\\ =f_{i}\left(q,t\right)+\frac{\rho_{r}I}{\kappa AG}\frac{\partial^{2}f_{i}\left(q,t\right)}{\partial t^{2}}-\frac{EI}{\kappa AG}\frac{\partial^{2}f_{i}\left(q,t\right)}{\partial q^{2}}\;\;\;\;\;\;\;\;\;\;\;\;\left(i=y,z\right), \end{array}$$ (3)

where *φ*_*i*_(*q*, *t*) denotes the *i*th component of the coordinate representation the centerline of the elastic tail with respect to the material frame of reference of the head in the **y**_r_-direction (*i* = *y*) and **z**_r_-direction (*i* = *z*), respectively, according to (1). In addition, *f*_*i*_(*q*, *t*) is the *i*th local fluid force acting at any arbitrary point along the tail, respectively. *I* is the second moment of area. Further, *ρ*_r_ and *A* are the density of the tail and the cross-section area, respectively. $\xi =\rho AR_{g}^{2}(1+\frac{E}{\kappa G})$, where *R*_g_, *κ*, and *G* are the radius of gyration, the Timoshenko corrections for circular cross section, and the shear modulus, respectively [31]. In (3), the local fluid forces, i.e., along tangent, normal, and bi-normal directions, are modeled using the RFT [29, 30] as follows:

$$\begin{array}{c} {\left[\begin{array}{ccc} f_{\text{t}} & f_{\text{n}} & f_{\text{b}} \end{array}\right]}^{\text{T}}=\left[\begin{array}{cc} RCR^{\text{T}} & -RCR^{\text{T}}S \end{array}\right]\left[\begin{array}{c} V_{\text{r}}\\ \Omega_{\text{r}} \end{array}\right].l \end{array}$$ (4)

where *f*_t_, *f*_n_, and *f*_b_ are the local forces along the local tangent, normal, and bi-normal directions throughout the elastic tail, as demonstrated in Fig 2. In (4), $C=\text{diag}\left[\begin{array}{ccc} C_{\text{t}} & C_{\text{n}} & C_{\text{b}} \end{array}\right]$, is a matrix of the resistive-force coefficients, where *C*_t_, *C*_n_, *C*_b_ denote the tangential, normal, and bi-normal local force coefficients of the tail in the local Frenet-Serret coordinates, respectively [29, 30], and are given by

$$\begin{array}{c} C_{t}=2\pi \mu (\ln(\frac{l_{t}}{r_{t}})-0.807)\;,\;C_{n}=C_{b}=4\pi \mu (\ln(\frac{l_{t}}{r_{t}})+0.193). \end{array}$$ (5)

In (4), **S** is a skew-symmetric matrix that denotes local cross product and is given by

$$\begin{array}{c} S=[\begin{array}{ccc} 0 & -\varphi_{z}\left(q,t\right) & \varphi_{y}\left(q,t\right)\\ \varphi_{z}\left(q,t\right) & 0 & -q\\ -\varphi_{y}\left(q,t\right) & q & 0 \end{array}]. \end{array}$$ (6)

The rotation matrix **R** between the local Frenet-Serret frame and the material frame of the robotic sperm is given by, $R=[\begin{array}{ccc} t & n & b \end{array}]$. Eq (4) projects the rigid-body velocity vector of the robotic sperm ${\left[\begin{array}{cc} V_{\text{r}} & \Omega_{\text{r}} \end{array}\right]}^{\text{T}}$ onto local Frenet-Serret frame, and predicts the local resistive-forces based on the resistive-coefficients (5). These forces are entered into (3) to calculate the instantaneous pattern throughout the elastic tail. The rigid-body velocity are calculated using the following equation of motion:

$$\begin{array}{c} {}[\begin{array}{c} F_{m}+F_{g}+F_{f}\\ T_{m}+T_{g}+T_{f} \end{array}]=0, \end{array}$$ (7)

where **F**_m_ and **T**_m_ are the magnetic force and magnetic torque exerted on the magnetic dipole of the robotic sperm, and **F**_g_ and **T**_g_ are the force and torque due to gravity and given by

$$\begin{array}{c} {}[\begin{array}{c} F_{m}\\ T_{m} \end{array}]=[\begin{array}{c} V(M\cdot \nabla )B\\ VM\times B \end{array}]\;\;,\;\;[\begin{array}{c} F_{g}\\ T_{g} \end{array}]=[\begin{array}{c} (m_{r}-m_{\text{disp}})R_{r}^{-1}g\\ SF_{g} \end{array}], \end{array}$$ (8)

where *V* is the volume of magnetic material embedded in the head. Further, *m*_r_ and *m*_disp_ are the mass of the robot and the displaced mass of the medium by the robot, respectively, **g** is the gravitational attraction vector, and **R**_r_ is the rotation matrix from robot’s frame of reference to laboratory frame of reference. In (7), **F**_f_ and **T**_f_ denote the following drag force and torque:

$$\begin{array}{c} {}[\begin{array}{c} F_{f}\\ T_{f} \end{array}]=[(\begin{array}{cc} D_{h} & -D_{h}S_{h}\\ S_{h}D_{h} & N_{h} \end{array})+\int_{0}^{l_{t}}(\begin{array}{cc} {\text{RCR}}^{T} & -{\text{RCR}}^{T}S\\ S{\text{RCR}}^{T} & -S{\text{RCR}}^{T}S \end{array})dl][\begin{array}{c} V_{r}\\ \Omega_{r} \end{array}], \end{array}$$ (9)

where the matrix **S**_h_ denotes local cross product as follows:

$$\begin{array}{c} S_{h}=[\begin{array}{ccc} 0 & -z_{\text{cov}} & y_{\text{cov}}\\ z_{\text{cov}} & 0 & -x_{\text{cov}}\\ -y_{\text{cov}} & x_{\text{cov}} & 0 \end{array}], \end{array}$$ (10)

where {*x*_cov_, *y*_cov_, *z*_cov_} denote the position of center of volume of the head with respect to the center of mass of the robotic sperm. Further, **D**_h_ and **N**_h_ are diagonal resistance matrices for blunt prolate spheroids and given by

$$\begin{array}{c} D_{h}=[\begin{array}{ccc} D_{x} & 0 & 0\\ 0 & D_{yz} & 0\\ 0 & 0 & D_{yz} \end{array}]\;\;\text{and}\;\;N_{h}=[\begin{array}{ccc} N_{x} & 0 & 0\\ 0 & N_{yz} & 0\\ 0 & 0 & N_{yz} \end{array}], \end{array}$$ (11)

where the resistive-coefficients *D*_*x*_ and *D*_*yz*_ are given by

$$\begin{array}{c} D_{x}=\frac{-4\pi \mu a}{\ln(\frac{2a}{b})-0.5}\;\;\text{and}\;\;D_{yz}=\frac{-8\pi \mu a}{\ln(\frac{2a}{b})+0.5}, \end{array}$$ (12)

where *a* and *b* are the major and minor diameters of the prolate spheroid head, respectively. In (9), the resistive coefficients *N*_*x*_ and *N*_*yz*_ are given by

$$\begin{array}{c} N_{x}=-\frac{16}{3}\pi \mu ab^{2}\;\;\text{and}\;\;N_{yz}=\frac{-\frac{8}{3}\pi \mu a}{\ln(\frac{2a}{b})-0.5}. \end{array}$$ (13)

Equations of motion (1)–(13) describe the elastohydrodynamics of our soft robotic sperm in a viscous fluid, and the theoretical predication is compared to experimental results.

## Results and discussions

We examine a group of robotic sperm samples suspended in an observation chamber containing silicone oil (Calsil IP 5.000, Caldic, Rotterdam, The Netherlands) with viscosities of 1 Pa.s and 5 Pa.s. The samples are observed using two microscopic systems to determine the swimming patterns from top- and side-view of the chamber, as shown in Fig 2. The chamber is fixed in the common center of an electromagnetic system with eight electromagnetic coils with the configuration shown in Fig 2 (See Experimental section). These coils exert the magnetic force and torque presented in (8) on the robots to achieve either planar or helical flagellar propulsion.

### Numerical scheme of the RFT-based model

The deformation of the tail and the drag forces along the tail are determined using finite-difference discretization (tail is discretized into 250 equally spaced mesh nodes). The partial differential Eq (3) are solved iteratively using Gauss-Seidel method. The time-dependent trajectory of the robotic sperm is calculated by forward Euler integration over consecutive time-steps (5 × 10^−5^ seconds). The velocity of the robotic sperm is obtained by solving (4)–(7) and averaging the results over 5 complete cycles of oscillating external magnetic fields. The minor and major diameter of the head of the robotic sperm are 21 μm and 56 μm, respectively. The length of the tail is 300 μm and its diameter is 3 μm.

The measured velocity of the robotic sperm is compared to the theoretical prediction of our model. It is also necessary to compare the deformation of the tail. However, our problem is an ‘initial condition problem’ that requires measuring the precise initial condition (position and orientation of the swimmer) and the initial transient of the electronics (the electromagnetic behavior of the driving system during startup). Therefore, the calculated time-dependent deformation along the tail will not be in a quantitative agreement with the measured deformations of the tail. In addition, geometric aberrations along the elastic tails of the fabricated robotic sperm samples via electrospinning hinders our effort to obtain quantitative agreement between the predicted time-dependent behaviour and measured deformations. Therefore, time-averaged velocity of the robotic sperm is only used to compare our theoretical predictions to experimental results.

### Planar flagellar propulsion

**Fig 3:**
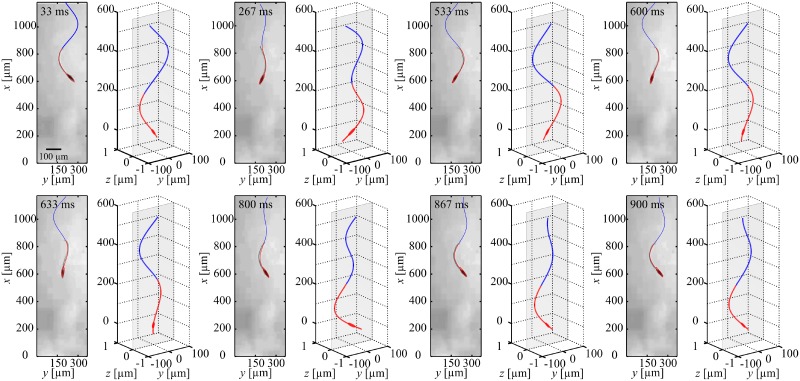
A robotic sperm achieves planar flagellar propulsion under the influence of a periodic magnetic field constrained in a plane. The robot swims at an average speed of 18.9 ± 0.5 μm/s (*n* = 5) in silicone oil with viscosity 1 Pa.s, at frequency of 1 Hz (S1 Dataset). The red curve indicates the real shape of the robot, while the blue curve is added to demonstrate the propagating wave to its full length (S1 Video).

**Fig 4:**
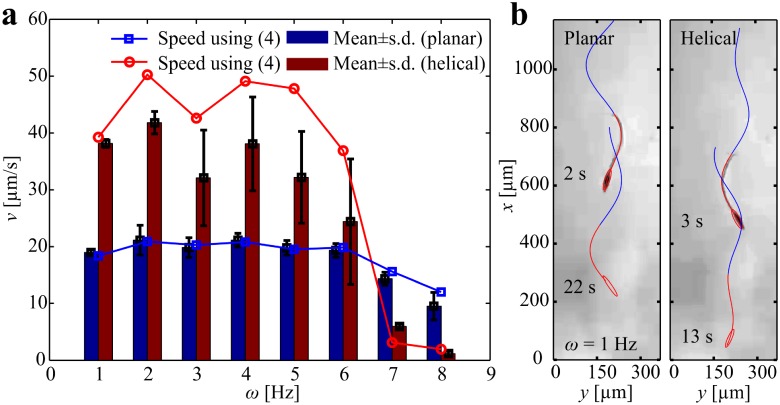
Frequency response of robotic sperm in silicone oil with viscosity of 1 Pa.s indicates the influence of the propulsion mode on the swimming speed. (a) Helical flagellar propulsion enables the robot to swim at higher speed than planar flagellar propulsion below actuation frequency of 6 Hz (S2 Video). (b) At actuation frequency *ω* = 1 Hz, a 358-μm-long robotic sperm swims at average speed of 18.9 ± 0.5 μm/s (*n* = 5) and 38.1±0.6 μm/s (n = 5) using planar and helical flagellar propulsion, respectively (S2 Dataset).

We achieve planar flagellar propulsion by applying in-plane periodic magnetic fields. These fields exert a magnetic torque to achieve wobbling of the head and induce a planar wave along the flexible tail. In Fig 3, we provide an experimental result of the robotic sperm during planar flagellar propulsion, at actuation frequency of 1 Hz. The head and flexible tail are indicated using the red curve, whereas the superimposed blue curve indicates the wave propagation along the tail. A 358-μm-long robotic sperm swims at an average speed of 18.9 ± 0.5 μm/s (*n* = 5) in silicone oil with viscosity of 1 Pa.s (S1 Video). Increasing the frequency of the oscillating magnetic fields enables the robotic sperm to swim at different speeds, as shown in Fig 4a. This frequency response indicates that the robot swims at an almost uniform speed at relatively low actuation frequencies. Above *ω* = 6 Hz, the speed of the robotic sperm decreases as we increase the frequency of the periodic magnetic fields (S2 Video). The frequency response of the robotic sperm also shows agreement with the theoretical prediction using our hydrodynamic model.

### Helical flagellar propulsion

**Fig 5:**
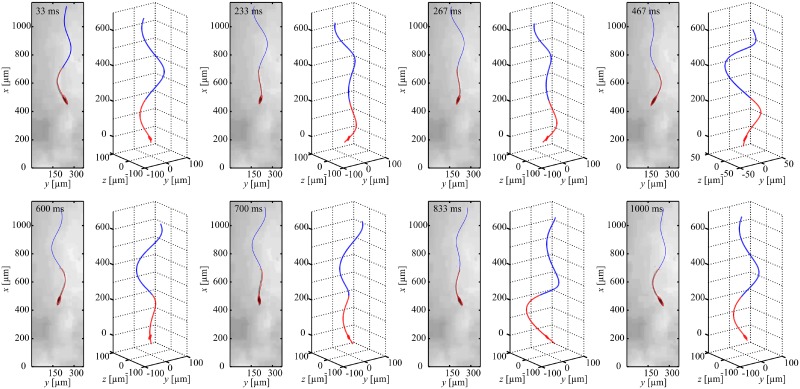
A robotic sperm achieves helical flagellar propulsion under the influence of out-of-plane magnetic field that follows a cone pointing in the direction of motion. The robot swims at an average speed of 38.1±0.6 μm/s in silicone oil with viscosity 1 Pa.s, at frequency of 1 Hz (S3 Dataset). The red curve indicates the real shape of the robot, whereas the blue curve is superimposed to represent the propagating wave along the flexible tail (S3 Video).

Now we turn our attention to helical flagellar propulsion by applying out-of-plane magnetic fields. These fields are generated such that the resultant magnetic field at the position of the head follows a cone pointing in the direction of motion. Fig 5 provides an experimental result of helical propulsion in silicone oil with viscosity 1 Pa.s, at actuation frequency of 1 Hz. The robot is indicated using the red curve, whereas the three-dimensional helical wave is superimposed using the blue curve to indicate the traveling wave. In this experiment, the robot swims at an average speed of 38.1 ± 0.6 μm/s (*n* = 5). Therefore, swimming using helical flagellar propulsion achieves an increase in the speed by factor of 2 compared to planar flagellar propulsion at the same actuation frequency (S3 Video). The frequency response of this swimming mode is also represented in Fig 4. The difference between the calculated and measured speeds are due to deviations between the nominal or measured parameters that are entered to the model such as the magnetic dipole moment, modulus of elasticity, rheological properties of the fluid, and dimensions of the robotic sperm. Nevertheless, our theoretical model provides qualitative agreement with the experiment and predicts optimal actuation frequencies at 2 Hz and 4 Hz. We also observe that helical propulsion enables the robot to swim at higher speed for a frequency range of 1 Hz to 6 Hz. Above *ω* = 6 Hz, planar flagellar propulsion is more efficient than helical flagellar propulsion.

### Switching between swimming modes

**Fig 6:**
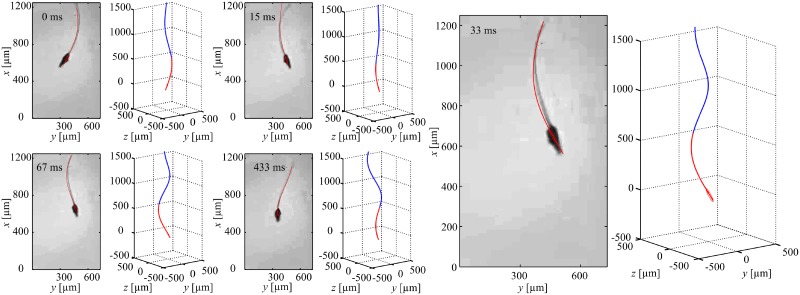
A robotic sperm switches between planar to helical flagellar propulsion, at frequency of 2 Hz. The speed of the robot is increased by a factor of 1.97 after the switch (S4 Dataset). The red curve indicates the real shape of the robot, whereas the blue curve is superimposed to represent the propagating wave along the flexible tail (S4 Video).

In order to mimic the unique ability of sperm cells to achieve planar-to-helical transition and conversation from helical-to-planar beating, we apply the two mentioned electromagnetic field patterns on the same robotic sperm to determine its response. Fig 6 indicates the configuration of the head and the flexible tail when the robot switches from planar-to-helical flagellar propulsion. We observe that a planar wave is induced along the flexible tail with amplitude of 90 μm and wave length of 800 μm. Switching to the out-of plane fields results in an switch to helical flagellar propulsion with a propagating wave with amplitude and wave length of 62 μm and 500 μm, respectively. This switch also results in an increase in the swimming speed by a factor of 1.97 (S4 Video).

### Influence of rheological properties of the medium

**Fig 7:**
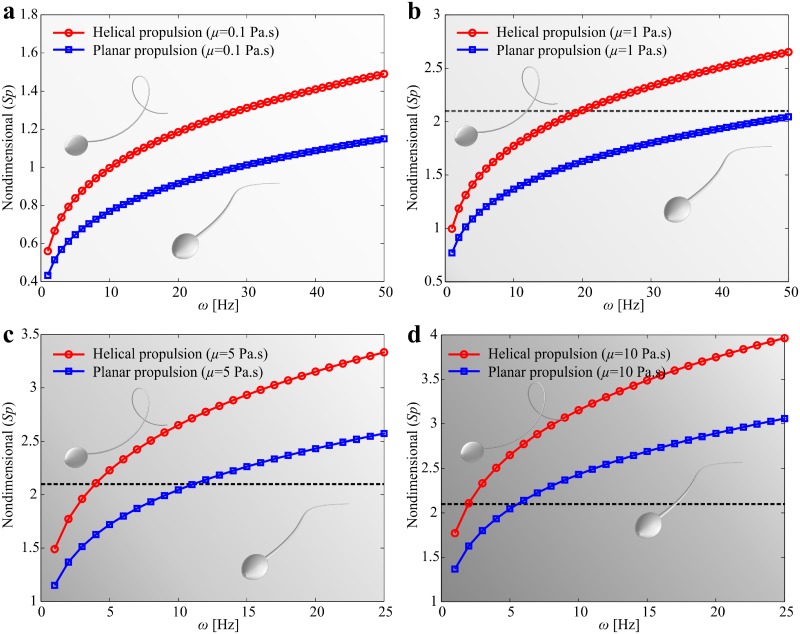
Influence of the viscosity of the background fluid on the swimming characteristic is determined for fluids with viscosities of 0.1 Pa.s, 1 Pa.s, 5 Pa.s, and 10 Pa.s. Helical flagellar propulsion is more efficient than planar flagellar propulsion at relatively low actuation frequency regardless to the viscosity of the fluid. (a) At low viscosity (*μ* = 0.1 Pa.s), optimal sperm number of 2.1 is approached at high actuation frequency (> 50 Hz). (b) At *μ* = 1 Pa.s, the optimal actuation frequencies are 20 Hz and 50 Hz for helical and planar flagellar propulsion, respectively. (c) At *μ* = 5 Pa.s, the optimal actuation frequencies are 4 Hz and 11 Hz for helical and planar flagellar propulsion, respectively. (d) Helical and planar flagellar propulsion have optimal swimming speed at 2 Hz and 6 Hz, respectively (S5 Dataset).

**Fig 8:**
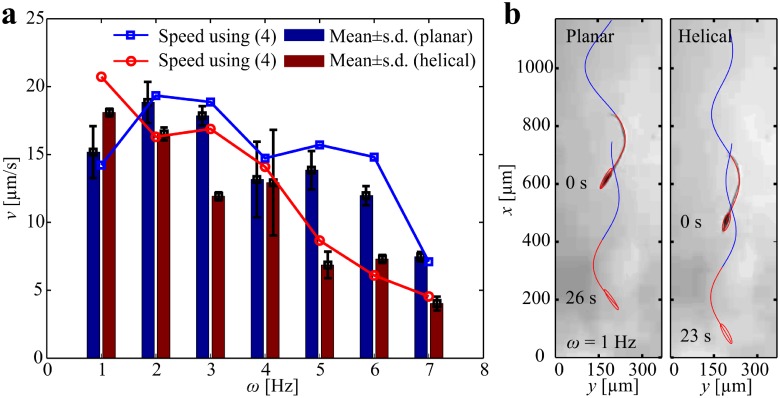
Frequency response of robotic sperm in silicone oil with viscosity of 5 Pa.s indicates the influence of the propulsion mode on the swimming speed. (a) Helical flagellar propulsion enables the robot to swim at higher speed than planar flagellar propulsion till actuation frequency of 2 Hz (S2 Video). (b) A 358-μm-long robotic sperm swimming at average speeds of 15.2 ± 2 μm/s (*n* = 5) and 18.1 ± 0.3 μm/s (*n* = 5) using planar and helical flagellar propulsion at *ω* = 1 Hz, respectively (S6 Dataset).

The central result from our hydrodynamic model is shown in Fig 7. We determine the sperm number (*Sp*) of the robotic sperm by varying the viscosity of the medium and the actuation frequency [33]. Sperm number is calculated using $Sp=l_{t}{\left(\frac{8\omega C_{\text{n}}}{r_{\text{t}}^{4}E}\right)}^{1/4}$ for planar flagellar propulsion and $Sp=l_{t}{\left(\frac{8\omega C_{\text{n}}}{r_{\text{t}}^{4}G}\right)}^{1/4}$ for helical flagellar propulsion, as shown in Fig 7. Planar wave propagation is confined to a plane, whereas helical wave propagation implies torsional stress in three-dimensional space which is governed by the shear modulus (*G*). Young’s modulus and Shear modulus are related with *E* = 2*G*(1 + *ν*), where *ν* is Poisson’s ratio. Therefore, we scale *Sp* with 2(1 + *ν*) for helical waves to indicate the difference between wave patterns. Sperm number of 2.1 indicates that the robotic sperm generates maximum propulsive force [34]. Therefore, at viscosity of 0.1 Pa.s, helical and planar flagellar propulsion necessitates relatively high actuation frequency to approach *Sp* = 2.1, as shown in Fig 7a. At viscosity of 1 Pa.s, helical flagellar propulsion approaches *Sp* = 2.1 at actuation frequency of 19.7 Hz, whereas planar flagellar propulsion approaches *Sp* = 2.1 above 50 Hz (Fig 7b). This behavior provides a qualitative agreement with our experimental frequency response (Fig 4a). The frequency response indicates that helical propulsion is more efficient below *ω* = 6 Hz for viscosity of 1 Pa.s. At viscosity of 5 Pa.s, helical and planar flagellar propulsion approach *Sp* = 2.1 at actuation frequencies of 4 Hz and 11.1 Hz, respectively. As the viscosity increases to 10 Pa.s, helical and planar flagellar propulsion approach the optimal sperm number at actuation frequencies of 2 Hz and 5.5 Hz, respectively. In order to further validate this observation, we determine the frequency response of the robotic sperm in silicone oil with viscosity of 5 Pa.s, as shown in Fig 8a. At low actuation frequency *ω* < 2 Hz, helical propulsion achieves greater swimming speed in a relatively high viscous medium. Fig 8b shows a 358-μm-long robotic sperm swimming at average speeds of 15.2 ± 2 μm/s (*n* = 5) and 18.1 ± 0.3 μm/s (*n* = 5) using planar and helical flagellar propulsion at *ω* = 1 Hz, respectively. At this actuation frequency, the speed of the robot during helical flagellar propulsion is greater than that of planar flagellar propulsion by a factor of 1.2. However, planar propulsion becomes more efficient as the actuation frequency increases (S4 Video).

## Conclusions

We demonstrate the ability of a soft robotic sperm to change its swimming speed controllably by tuning the actuation frequency and its propulsion modes. This capability enables biological sperm cells to maintain high swimming speed regardless of the rheological properties of the background fluid. Our experimental results and theoretical predictions show that helical flagellar propulsion is more efficient than planar flagellar propulsion for relatively low actuation frequencies. In silicone oil with viscosity of 1 Pa.s, helical flagellar propulsion is twice as fast as planar flagellar propulsion for relatively low actuation frequencies, and in silicone oil with viscosity of 5 Pa.s, helical propulsion achieves higher speed than planar propulsion by a factor of 1.2 for low actuation frequencies. Similar to their biological counterparts, soft robotic sperm has to switch from helical-to-planar flagellar propulsion to avoid relatively large reduction in speed at high actuation frequencies. The ability to switch between propulsion modes to maintain relatively high swimming speed is essential for navigation in bodily fluids (with different viscosities) to perform targeted diagnosis, targeted therapy, and broad biomedical applications [35, 36]. Our work provides the necessary theoretical and experimental frameworks to design soft microrobots capable of adapting to a given medium with variable viscosity.

## Experimental section

**Robotic sperm preparation:** The robotic sperm samples are fabricated using electrospinning. The samples are prepared using polystyrene with concentration of 17 weight % in dimethylformamide. Magnetic particles (45-00-252 Micromod Partikeltechnologie GmbH, Rostock-Warnemünde, Germany) are used to provide magnetic dipole to the samples. The weight ratio of the nanoparticles to polystyrene is 3:2, and the electric field potential is adjusted to be 150 kV/m. The solution is pumped at flow rate of 1 mL/h and beaded fibers are collected and their dimensions are determined using scanning electron microscopy images (Fig 1a). The modulus of elasticity of the tail is measured using depth sensing indentation as 0.58 ± 0.05 GPa [37].

**Magnetic field control:** Swimming of the robotic sperm is studied in silicone oil (Calsil IP 5.000, Caldic, Rotterdam, The Netherlands) of viscosity 1 Pa.s and 5 Pa.s in a deep chamber of 2 cm depth using a microscopic unit (MF Series 176 Measuring Microscopes, Mitutoyo, Kawasaki, Japan). Videos are acquired using a camera (avA1000-120kc, Basler Area Scan Camera, Basler AG, Ahrensburg, Germany) and a 10× Mitutoyo phase objective. The chamber is contained in the common center of 8 electromagnetic coils (Fig 2) that are supplied with input currents independently [33]. Each electromagnetic coil (inner-diameter 20 mm, outer-diameter 40 mm, and length 80 mm) has 3200 turns and thickness of the wire is 0.7 mm, and generates maximum magnetic field of 70 mT (using current input of 4 A) in the workspace. The field is measured using a calibrated three-axis digital Teslameter (Senis AG, 3MH3A-0.1%-200 mT, Neuhofstrasse, Switzerland).

**Frequency response characterization:** Two magnetic field patterns are used to achieve planar and helical flagellar propulsion. Uniform in-plane magnetic field along direction of motion with sinusoidally varying orthogonal component is used to achieve planar wobbling of the magnetic head and induce planar wave propagation along the flexible tail. Electromagnetic coils number 1, 3, 5, and 7 (Fig 2) are supplied with control signal $\sin\Omega_{z}+\frac{\pi }{4}\cos\left(2\pi \omega t\right))$, while coils number 2, 4, 6, 8 are provided with $\cos\Omega_{z}+\frac{\pi }{4}\cos\left(2\pi \omega t\right))$. On the other hand, helical wave propagation is achieved by out-of-plane wobbling of the magnetic head. Coils 1 and 7 are supplied with max(0, sin(2*πωt* − *π*)), whereas coils 2 and 8 are supplied with $\max(0,\sin\left(2\pi \omega t-\frac{\pi }{2}\right))$. Coils 3 and 5 are provided with $\max(0,\sin\left(2\pi \omega t+\frac{\pi }{2}\right))$ and coils 4 and 6 are supplied with max(0, sin(2*πωt*)). Frequency response of the soft robotic sperm is determined by varying the actuation frequency *ω* throughout a frequency range of 1 Hz to 8 Hz. The average speed of the robotic sperm is calculated using 5 trials, at each frequency.

## Supporting information

S1 Video A robotic sperm achieves planar flagellar propulsion under the influence of a periodic magnetic field constrained in a plane. The robot swims at an average speed of 18.9 ± 0.5 μm/s (*n* = 5) in silicone oil with viscosity 1 Pa.s, at frequency of 1 Hz.
(MP4)

S2 Video Frequency response of robotic sperm in silicone oil with viscosity of 1 Pa.s indicates the influence of the propulsion mode on the swimming speed. Helical flagellar propulsion enables the robot to swim at higher speed than planar flagellar propulsion below actuation frequency of 6 Hz.
(MP4)

S3 Video A robotic sperm achieves helical flagellar propulsion under the influence of out-of-plane magnetic field that follows a cone pointing in the direction of motion. The robot swims at an average speed of 38.1 ± 0.6 μm/s in silicone oil with viscosity 1 Pa.s, at frequency of 1 Hz.
(MP4)

S4 Video A robotic sperm switches between planar to helical flagellar propulsion, at frequency of 2 Hz. The speed of the robot is increased by a factor of 1.97 after the switch.
(MP4)

S1 Dataset Deformation of the tail is measured during planar flagellar propulsion. The dataset provides the deformation of the tail at different time instants.
(ZIP)

S2 Dataset Frequency response of a robotic sperm in silicone oil with viscosity of 1 Pa.s. The dataset provides the average velocity of the robotic sperm versus the actuation frequency.
(ZIP)

S3 Dataset Deformation of the tail is measured during helical flagellar propulsion. The dataset provides the deformation of the tail at different time instants.
(ZIP)

S4 Dataset Deformation of the tail is measured during transition between planar and helical flagellar propulsion. The dataset provides the deformation of the tail at different time instants.
(ZIP)

S5 Dataset Sperm number is calculated to show the influence of the viscosity on the planar and helical flagellar propulsion. The dataset provides calculated sperm number for various viscosities.
(ZIP)

S6 Dataset Frequency response of a robotic sperm in silicone oil with viscosity of 5 Pa.s. The dataset provides the average velocity of the robotic sperm versus the actuation frequency.
(ZIP)
